# Lifestyle Modifications for Migraine Management

**DOI:** 10.3389/fneur.2022.719467

**Published:** 2022-03-18

**Authors:** Mendinatou Agbetou, Thierry Adoukonou

**Affiliations:** ^1^Department of Neurology, University of Parakou, Parakou, Benin; ^2^Clinic of Neurology, Teaching Hospital of Parakou, Parakou, Benin; ^3^Inserm U1094, IRD U270, Univ. Limoges, CHU Limoges, EpiMaCT - Epidemiology of Chronic Diseases in Tropical Zone, Institute of Epidemiology and Tropical Neurology, OmegaHealth, Limoges, France

**Keywords:** migraine, lifestyle modification, physical activity, obesity, diet

## Abstract

Migraine is a disabling disease that inflicts a heavy burden on individuals who suffer from it. Significant advances are being made in understanding the pathophysiology and treatment of the disease. The role of lifestyle modifications has become increasingly predominant. We reviewed the current and available data on the role of a healthy lifestyle in the management of migraine. Physical activity, management of obesity, a healthy diet, and a better lifestyle, such as adequate sleep and avoidance of drug abuse, significantly contribute to reducing the frequency and severity of attacks. It is important to consider these factors in the overall management strategies for migraine sufferers.

## Introduction

The Global Burden of Disease study in 2016 identified migraine as the second leading cause of years lived with disability (1) with an age-standardized disability-adjusted life years rate of 596.8 per 100,000 (2). The number of productive days at work was reduced by half or more as headache was significantly higher in occurrence in migraine sufferers (3). The direct and indirect costs and their impact on family, social, and professional life are also high (4, 5). The estimated cost of productivity loss associated with presenteeism (absenteeism) due to migraine was calculated at 21.3 billion US$/year in some studies (3). Episodic migraine can progress to chronic migraine, and about 3% of patients with episodic migraine report a very severe headache-related disability, as defined by the Migraine Disability Assessment Scale (6). Approximately 25% of individuals with chronic migraine have headache-related disability (6). Many risk factors contribute to chronicity and increase in episodic migraine frequency (7), some of which are modifiable, such as overuse of acute migraine medication, obesity, metabolic syndrome, depression, and stressful life events (8, 9). These risk factors may serve as targets for future preventive interventions. Even if the results are not unanimous, several publications have confirmed the effectiveness of reducing the burden of migraine with changes in the lifestyles of migraine sufferers (10). Migraine preventive therapy helps reduce the frequency of migraine attacks, days with migraine and headache, severity of symptoms, frequency of acute migraine therapy, and migraine-related disability (11). Here, we review the role of lifestyle changes and their benefits in managing migraine.

## Methods

This was a general mini-review and not a systematic review. For this general mini-review, the research was conducted in three electronic databases (PubMed, ISI Web of Science, and Google scholar). The keywords used were migraine, lifestyle, alcohol, obesity, overweight, caffeine, physical activity, smoking, diet, hydration, depression, insomnia, and drug abuse. Only articles published between January 2000 and May 2021 in French or English were included. We also used other articles from gray literature.

## Migraine and Lifestyle Management

**Figure 1:**
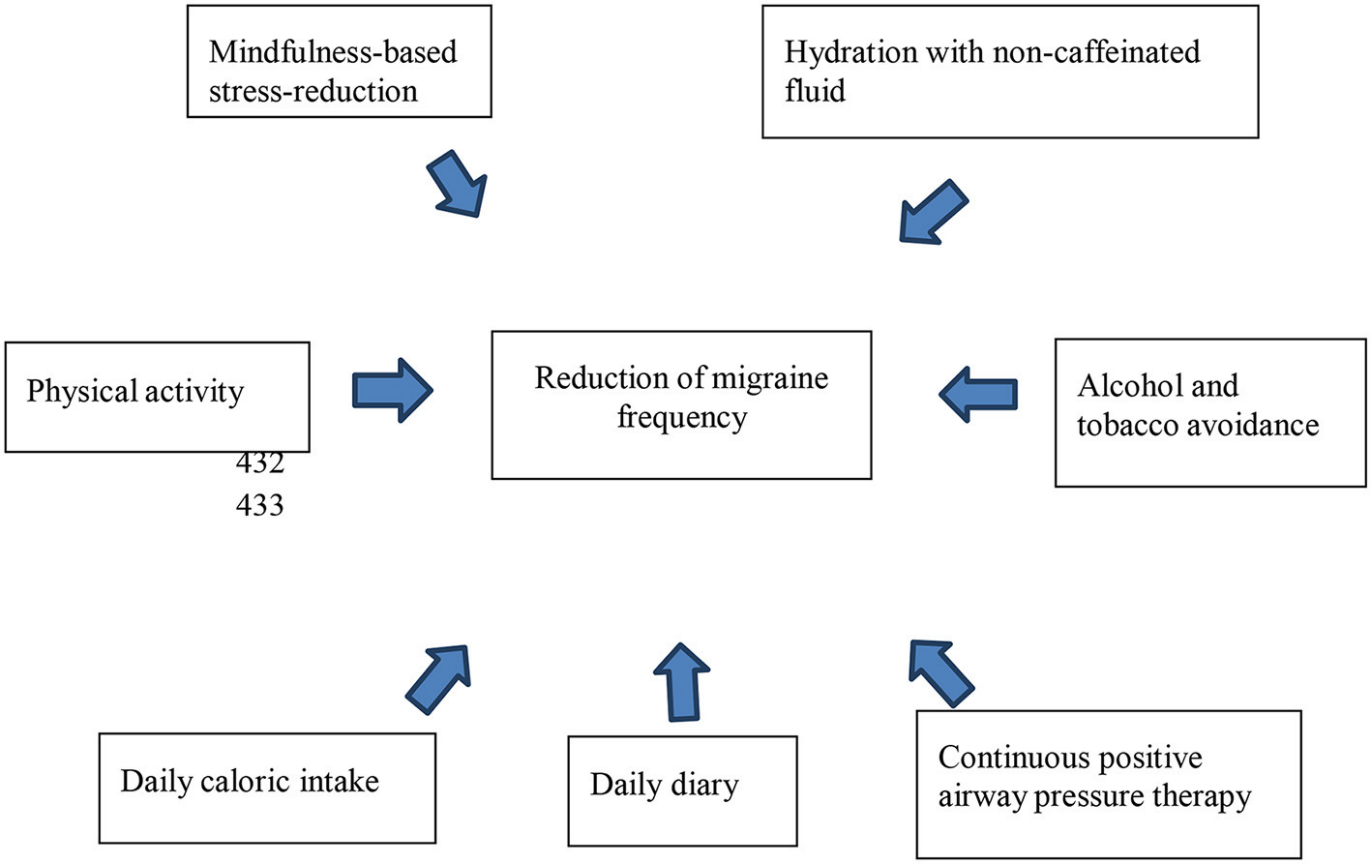
Lifestyle modification in migraine management.

In addition to migraine attack trigger identification and avoidance, avoidance of risk factors for developing more frequent migraine attacks through a change in lifestyle is an important part of preventive measures for migraine (Figure 1). It has no side effects and is indicated for all migraine sufferers at a low cost with little risk to the patient (12). The acronym SEED, which means Sleep, Exercise, Eat, and Diary, was proposed in a recent update to summarize the lifestyle changes needed to improve migraine (10). Non-pharmacological treatments have been shown to be effective in controlling migraine (13, 14). Regular lifestyle behavior helps to control chronic migraine as patients without regular lifestyle behaviors are more likely to have chronic migraine than episodic migraine (12).

### Physical Activity

Obesity, defined as a body mass index (BMI) [weight (kg)/height2 (m2)] ≥ 30, is associated with an increased frequency and severity of attacks among patients with episodic migraine (15). The interrelationships between migraine frequency and obesity are not well-known, but a bidirectional link between the two conditions has been noted (16). Migraine is significantly associated with obesity and being overweight, whereas the clinical features of migraine are not associated with BMI (15). Meta-analysis of the available observational studies suggested an association between migraine and obesity that is likely mediated by sex and migraine frequency (17, 18). In addition, BMI category has a consistent and increasing relationship with transformed migraine prevalence (16), and chronic migraine was associated with insulin resistance status, particularly when it is combined with obesity (8). In obese migraine patients, hypothalamic deregulation leads to alterations in peptides, neurotransmitters, and adipocytokines involved in energy homeostasis and regulation of feeding, especially via the orexinergic system (19, 20). Serotonin or orexins A or B can influence food intake, along with the feeling of satiety and modulation of nociceptive messages. Another hypothesis highlights the possible role of increased intracranial pressure found in a proportion of obese patients and some migraine patients (21). Pro-inflammatory mediators, including IL-1, IL-6, tumor necrosis factor-α, and calcitonin gene-related peptide (CGRP), play an important role in the pathophysiological mechanisms of these two conditions (22, 23). Likewise, an inflammatory state, induced by leptin and adiponectin secreted by adipose tissue, exists in obesity, and actively contributes to increasing migraine frequency or migraine transformation (24). Overactivation of the reward circuitry in obesity can lead to food addiction and/or excessive eating behaviors (25). In addition, one of the side effects of some migraine medications is weight gain (26).

Low levels of physical activity are associated with an increased migraine frequency (27). Therefore, weight loss may be proposed to reduce headache frequency and severity (28). Several strategies can be used to achieve this goal, including behavioral weight loss, pharmacotherapy, and bariatric surgery. However, weight loss is the recommended first-line treatment (29). Behavioral therapy in diet and physical activity interventions are more widely available and are recommended as primary intervention strategies. Outside of migraine attacks, internet-based resources, physical groups, or individual activities encourage physical activity in migraine sufferers (27). Approximately 150–300 min of moderate-intensity aerobic exercise per week (30) and increased lifestyle activities, such as walking the dog, parking farther away, or taking the stairs are encouraged. An aerobic exercise program that included relaxation had a similar effect to topiramate in reducing migraine pain intensity and frequency (31). Moreover, activities such as walking, jogging, cross-training, and cycling also have beneficial effects when completed for 30–60 min, 3–5 times a week. Physical activity has a positive cross-sectional effect on most of the modifiable risk factors of migraine and can improve the patients' quality of life (32, 33). Apart from weight loss, stress reduction, decreased anxiety, depressed mood, and depression, physical activity improves sleep efficiency and sleep quality, thereby inducing deep sleep, reducing daytime sleepiness, and decreasing the frequency of medication use to aid sleep (30).

### Diet

Diet is an important lifestyle aspect. However, there is no specific diet for migraine sufferers. For individuals who are obese, a weight-loss diet is recommended. The total daily caloric intake of between 1,200 and 1,500 calories for women and 1,500 to 1,800 calories for men can be adjusted to induce weight loss (29). Several diets can improve migraine symptoms such as the following: elimination diets, diets high in certain nutrients, and epigenetic diets. Diet strategies, such as low fat, low carbohydrate, and high protein diet, can result in weight loss and similar cardiovascular benefits (29). Elimination diets require the identification of provocative dietary ingredients and their subsequent elimination (34). One example is the gluten-free diet among patients with celiac disease, which decreases headache or migraine frequency from 51.6 to 100% (35). Other elimination diets, such as immunoglobulin G-elimination, antihistamine, tyramine-free, and low-fat diets have contradictory results and might cause malnutrition in cases of total avoidance (36–38). Moreover, low-glycemic index diets showed improvement in migraine frequency in a diet group and in a control group of patients who took a standard migraine-preventive medication (39). On the other hand, diets high in certain food or ingredient ratios can also provide satisfactory results. Diets containing high levels of omega-3 fatty acids and low levels of omega-6 fatty acids reduce the duration and frequency of migraine (40, 41). Ketogenic diet in overweight individuals, low-sodium diet in pre-hypertensive patients or the elderly population, and a high-sodium diet among young women without hypertension and with a low-to-normal BMI or who have postural tachycardia syndrome may be beneficial (42, 43). Neuroprotection, improvement in mitochondrial function, compensation for serotonergic dysfunction, decrement in CGRP levels, and suppression of neuroinflammation are the main mechanisms of action of these diets (42, 43). An epigenetic diet that can target DNA methylation, such as a folate-rich diet (44), modified Atkins, and Mediterranean diet, has also been reported (41). Some studies have demonstrated that migraine is more common when meals are skipped, particularly breakfast (45, 46). Thus, the standard advice for migraine sufferers is to take meals at regular intervals. In all cases, an appropriate diet selected by physicians and dietitians is recommended to ensure the psychosocial well-being of migraine sufferers.

### Alcohol and Smoking

Alcohol is a trigger for migraine attacks in 75% of patients (47) through an inflammatory pathophysiologic mechanism (48). Other mechanisms may be involved, including vasodilatory effects, dehydration, toxicity, histamine, tyramine, sulfites, flavonoids, and 5-HT release (48). At this step, red wine is most indexed. However, all forms of alcohol may be trigger factors (49).

The pathogenesis of smoking or the use of nicotine in migraine onset is controversial and their action is direct on the central nervous system (50). Migraine attacks can be triggered by smoking (51). Particularly, if the total number of cigarettes smoked exceeds 5/day, it could subjectively precipitate a migraine attack (52). Among former smokers, smoking cessation is recommended (50).

### Hydration

Headache is associated with fluid restriction and dehydration (53). A decrease in blood volume would result in poor oxygenation of the brain (54). Increased hydration status in migraine leads to a balanced plasma osmolality and ion concentrations and can improve migraine (55). The recommended amount of water intake is not well-known. In some studies, it varies from 1.8 to 4 L per day (56, 57). According to the Institute of Medicine, daily water intake is a function of age and gender and median total water intake for adults ranges from 2.7 to 3.7 L with extremes of 1.4 to 6.2, or 9–13 cups per day (58).

### Caffeine and Migraine

Caffeine is an adenosine receptor antagonist with reversible effects on migraine. Its regular use is a risk factor for more frequent headaches. In addition, caffeine withdrawal can also induce headaches (59–61). There is a dose-dependent risk of headache, with a prevalence of 6.3 (62) to 14.5% (63).

Therefore, limiting caffeine consumption per day or discontinuation of caffeine consumption has been suggested.

### Psychiatric Comorbidity

A stressful lifestyle is linked to both the onset of migraine attacks and weight gain. Anxiety and depression are psychiatric comorbidities and risk factors for migraine, with higher odds of anxiety than depression (64). Depression is a significant predictor of chronic migraine onset (OR = 1.65, 95% CI 1.12–2.45) with a depression-dose effect (9). They share a bidirectional relationship where major depression increases the risk of migraine and migraine increases the risk for major depression (65). Cognitive-behavioral therapy in individualized sessions or group sessions, either in person or online, improves mental status, impacts weight loss, and decreases migraine symptoms (66). Mindfulness-based stress-reduction programs can reduce pain intensity, headache frequency, and disability and improve self-efficacy and quality of life by encouraging pain acceptance (67, 68). In addition, relaxation techniques are used in migraine management, leading to progressive muscle relaxation and adequate deep breathing techniques (69). All these methods can be combined to reduce the morbidity of migraine.

### Sleep Comorbidities

Lower quality of life, increased stress levels, and psychiatric comorbidities have been highlighted among migraine sufferers with sleep disorders (70). In addition, the risk of developing migraine in adults with sleep-related breathing disorders has increased (71). Poor sleep is a migraine trigger in which sleep apnea and insomnia are associated with migraine burden—symptoms that must be screened (72). Other common sleep disorders include short sleep duration, snoring, sleep-related breathing disorders, and restless leg syndrome (71, 73, 74). Insomnia prevalence among subjects with probable migraine is higher than in non-headache controls with a headache frequency. Similarly, Headache Impact Test-6 scores were also significantly higher in migraine sufferers with insomnia than in those without insomnia (75). Sleep is an effective treatment for migraine attacks (11) and there is a sleep hygiene benefit in chronic migraine, including keeping the bedroom quiet, dark, and cool; keeping the bed for sleep only; avoiding phones, tablets, or television in the bedroom; and having a regular bedtime (76, 77).

Insomnia can be managed by sleep and restriction of naps (78), while continuous positive airway pressure therapy for sleep apnea reduces the frequency of migraine (79).

### Diary and Migraine Applications and Devices

A regular electronic diary of attacks is considered superior to a paper diary (80). Currently, there is a quartet of cellphone medication adherence apps (81). These can track triggers, duration, frequency, topography, type, and associated signs to better characterize migraine. Many apps and devices integrated into smartphones are currently available and provide an electronic daily diary for headaches (82), leading to better management of migraine and better adherence to treatment (83). In addition, thanks to the applications, patients stay connected with a community of other patients suffering from migraine, thereby improving their stress and low mood (84).

Drug overuse is a major risk factor for migraine chronicity and an increase in headache frequency (24). A daily headache diary can help the evaluation for acute migraine medication use and allow management or discontinuation of such drugs.

## Conclusion

Migraine triggers are numerous. Lifestyle modifications and avoidance of triggers are essential in reducing the frequency and severity of migraine attacks. Managing obesity, alcohol, and tobacco consumption discontinuation, regular physical activity, sufficient hydration, and a healthy lifestyle are highly accessible and cost-efficient interventions for any patient with migraine. Nevertheless, large cohort follow-up studies on this population are warranted to obtain more information on environmental and lifestyle factors.

## Author Contributions

MA was involved in acquisition of data and drafted the manuscript for intellectual content. TA was involved in design and conceptualization of the study and final approval of the version to be published.

## Conflict of Interest

The authors declare that the research was conducted in the absence of any commercial or financial relationships that could be construed as a potential conflict of interest.

## Publisher's Note

All claims expressed in this article are solely those of the authors and do not necessarily represent those of their affiliated organizations, or those of the publisher, the editors and the reviewers. Any product that may be evaluated in this article, or claim that may be made by its manufacturer, is not guaranteed or endorsed by the publisher.
